# MetaboLights: open data repository for metabolomics

**DOI:** 10.1093/nar/gkad1045

**Published:** 2023-11-16

**Authors:** Ozgur Yurekten, Thomas Payne, Noemi Tejera, Felix Xavier Amaladoss, Callum Martin, Mark Williams, Claire O’Donovan

**Affiliations:** European Molecular Biology Laboratory, European Bioinformatics Institute (EMBL-EBI), Wellcome Genome Campus, Hinxton, Cambridge CB10 1SD, UK; European Molecular Biology Laboratory, European Bioinformatics Institute (EMBL-EBI), Wellcome Genome Campus, Hinxton, Cambridge CB10 1SD, UK; European Molecular Biology Laboratory, European Bioinformatics Institute (EMBL-EBI), Wellcome Genome Campus, Hinxton, Cambridge CB10 1SD, UK; European Molecular Biology Laboratory, European Bioinformatics Institute (EMBL-EBI), Wellcome Genome Campus, Hinxton, Cambridge CB10 1SD, UK; European Molecular Biology Laboratory, European Bioinformatics Institute (EMBL-EBI), Wellcome Genome Campus, Hinxton, Cambridge CB10 1SD, UK; European Molecular Biology Laboratory, European Bioinformatics Institute (EMBL-EBI), Wellcome Genome Campus, Hinxton, Cambridge CB10 1SD, UK; European Molecular Biology Laboratory, European Bioinformatics Institute (EMBL-EBI), Wellcome Genome Campus, Hinxton, Cambridge CB10 1SD, UK

## Abstract

MetaboLights is a global database for metabolomics studies including the raw experimental data and the associated metadata. The database is cross-species and cross-technique and covers metabolite structures and their reference spectra as well as their biological roles and locations where available. MetaboLights is the recommended metabolomics repository for a number of leading journals and ELIXIR, the European infrastructure for life science information. In this article, we describe the continued growth and diversity of submissions and the significant developments in recent years. In particular, we highlight MetaboLights Labs, our new Galaxy Project instance with repository-scale standardized workflows, and how data public on MetaboLights are being reused by the community. Metabolomics resources and data are available under the EMBL-EBI’s Terms of Use at https://www.ebi.ac.uk/metabolights and under Apache 2.0 at https://github.com/EBI-Metabolights.

## Graphical Abstract

**Graphical Abstract:**
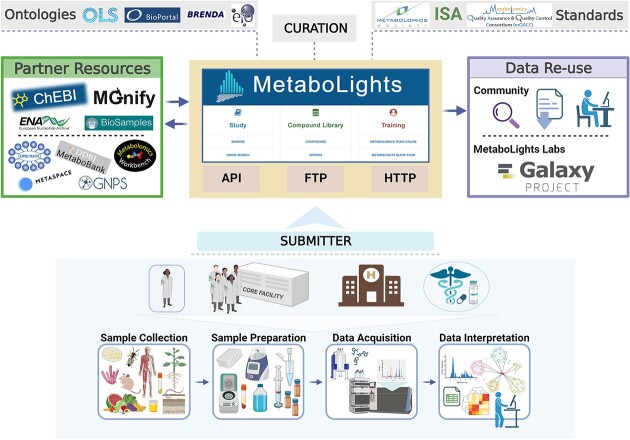

## Introduction

Metabolomics is the systematic study of the small molecular metabolites in a cell, tissue, biofluid or culture that are the tangible result of cellular processes or environmental stimuli. Collectively, these metabolites and their interactions within a biological system are known as the metabolome. Just as genomics is the study of DNA and genetic information within a cell, metabolomics is the study of substrates and products of metabolism, which are influenced by both genetic and environmental factors (1,2). Because metabolites and their concentrations, unlike other ’omics measures, directly reflect the underlying biochemical activity and state of cells/tissues, metabolomics best represents the molecular phenotype. Integrating metabolomics allows more confident interpretation of the other ’omics, and it is increasingly used in basic and applied biological research in areas such as systems biology and metabolic modelling, pharmaceutical research, nutrition and toxicology.

Our challenge is to capture the growing amount, depth and diversity of metabolomics data and make them easily available and interpretable, and integrated with the wider ’omics community, for the benefit of our users. We describe the significant developments that have been made with a focus on how we are positioning MetaboLights (3,4) to address its increasing use and importance in biosciences.

## Progress and developments

### Curation and content

Since the first release in 2012, MetaboLights has experienced year-on-year growth as the metabolomics community embraces the value and impact of open data policies. Registered accounts span 97 countries across Europe (32), Oceania (4), Americas (14), Asia (33) and Africa (13). There were 8544 studies as of September 2023 in MetaboLights, compared to 1432 studies in January 2020. The average number of studies per month was 218 for the first 6 months in 2023. In total, 1358 were ‘public’ (open and available to use), 98 were ‘in review’ (processed but under embargo), 863 were ‘in curation’ (awaiting review by MetaboLights) and 6396 were ‘submitted’ (preparation ongoing by the user). The latter statistic reflects the growing importance of MetaboLights as a platform for storing data while studies are in progress and for the processing of the data themselves. For public studies, this equates to 270,403 samples, 2,761 assays, 439,537 data files and 1,687,165 metabolites/unknowns/features. Data hosted on MetaboLights, 128+ terabytes in total, are across 6,815 different organism/organism parts, with a significant proportion of studies now human-based, *Homo sapiens* (blood plasma, blood serum, urine), followed by model organisms such as *Mus musculus* (liver, blood plasma, blood serum) and *Arabidopsis thaliana* (leaf, rosette leaf, root). Untargeted data are more prevalent than targeted, mass spectrometry (MS)—that is, liquid chromatography (LC), gas chromatography and direct infusion (DI)—more prevalent than nuclear magnetic resonance (NMR) spectroscopy and unassigned features more prevalent than assigned metabolites (with a database identifier). Study publications link to a variety of journals from Nature Publishing Group (*Nature Communications*), Frontiers Media SA (*Frontiers in Microbiology*), Multidisciplinary Digital Publishing Institute (*Metabolites*), BMC (*BMC Bioinformatics*) and PLOS (*PLoS One*).

The metabolomics field evolves constantly—this is reflected in the metabolomics data deposited to repositories such as MetaboLights. In MetaboLights, for example, the numbers of reported samples, assays and metabolites in studies have increased. Complex data, that is, intricate quality assurance/quality control (5), advanced acquisition (MS2 acquisition, collision cross-section, ion mobility, etc.) and multi-omics design, have become more prevalent. MTBLS718 (https://www.ebi.ac.uk/metabolights/MTBLS718) deposited by the UK National Phenome Centre (6), for example, encompasses specified ‘sample type’ and ‘assay role’ parameters to delineate concepts such as ‘pooled quality control sample’ (‘study reference’) and ‘legacy experimental control sample’ (‘long-term reference’) (7). The former is now an adopted characteristic for samples in MetaboLights. Boundaries continue to be pushed by vendors also with new instrumentation/technology—now LC–MS studies often include both MS1 and MS2 data such as data-dependent/information-dependent acquisition or data-independent acquisition, MTBLS2207 (https://www.ebi.ac.uk/metabolights/MTBLS2207) and MTBLS1108 (https://www.ebi.ac.uk/metabolights/MTBLS1108), respectively, for example. Efforts to streamline multi-omics integration have continued through involvement with consortia such as HoloFood and the HoloFood Data Portal (https://www.holofooddata.org), where metabolomics data in MetaboLights (MTBLS6988, https://www.ebi.ac.uk/metabolights/MTBLS6988) are linked using BioSamples (https://www.ebi.ac.uk/biosamples) (8) at the sample level with genomic data at the European Nucleotide Archive (https://www.ebi.ac.uk/ena) (9) and metagenomics data at MGnify (https://www.ebi.ac.uk/metagenomics) (10). This is an illustrative example of the positive collaboration between the EMBL-EBI resources and our wider user communities.

The MetaboLights team with its expert curation and ontology development continues to collaborate to develop standards and improve reporting, adopting the ISA (investigation, study and assay) model/standard (11) and aligning to the FAIR (findability, accessibility, interoperability and reusability) principles (12). Notable examples from the metabolomics community include ‘Quality assurance and quality control reporting in untargeted metabolic phenotyping: mQACC recommendations for analytical quality management’ (13), ‘Grapevine and wine metabolomics-based guidelines for FAIR data and metadata management’ (14) and ‘Progress towards an OECD reporting framework for transcriptomics and metabolomics in regulatory toxicology’ (15). Moreover, GNPS-MassIVE (https://gnps.ucsd.edu) introduced ReDU (16) to concur with ISA for MetaboLights, mwTab for Metabolomics Workbench (https://www.metabolomicsworkbench.org) and MAGE-TAB for MetaboBank (https://www.ddbj.nig.ac.jp/metabobank).

### Data reuse

Reuse of data deposited to MetaboLights often starts during curation, where metabolite names are assigned a database identifier with ChEBI (Chemical Entities of Biological Interest) (https://www.ebi.ac.uk/chebi) (17). Metabolites not yet in ChEBI are prepared with information related to chemistry, biology and source, and submitted by MetaboLights (36 478 entries to date). This enables aggregation of metadata, for example, ‘characteristics’, ‘factor value’ and ‘parameter value’, as found in the Compound Library on MetaboLights. ChEBI is used by biological databases worldwide, which facilitates integration with other ’omics also.

GNPS has been at the forefront of data reuse in metabolomics—now with the GNPS Dashboard (https://dashboard.gnps2.org) (18), through the Universal Spectrum Identifier (19), data from MetaboLights (among others) can be viewed directly with a web browser, for example, QC07.mzML from MTBLS1124 (https://www.ebi.ac.uk/metabolights/MTBLS1124, https://dashboard.gnps2.org/?usi=mzspec:MTBLS1124:QC07.mzML), and even processed with tools such as MZmine 2 (20). The same means can be used to perform classical molecular networking. Data from MetaboLights can also be found as part of MASST (https://masst.gnps2.org), the Mass Spectrometry Search Tool, which enables searches of small molecule tandem MS data in public repositories (21).

Across literature are multiple examples where the metabolomics community has similarly leveraged data public on MetaboLights to develop new methods/tools, across preprocessing, statistical analysis and metabolite identification (MTBLS797, https://www.ebi.ac.uk/metabolights/MTBLS797, and MTBLS709, https://www.ebi.ac.uk/metabolights/MTBLS709) (22,23), or build new workflows (MTBLS28, https://www.ebi.ac.uk/metabolights/MTBLS28) (24), as well as create specific resources for *Drosophila*, tomato fruits, interchemical correlations, etc. (MTBLS36, https://www.ebi.ac.uk/metabolights/MTBLS36, MTBLS417, https://www.ebi.ac.uk/metabolights/MTBLS417, MTBLS136, https://www.ebi.ac.uk/metabolights/MTBLS136, MTBLS204, https://www.ebi.ac.uk/metabolights/MTBLS204, and MTBLS205, https://www.ebi.ac.uk/metabolights/MTBLS205) (25–29), and how-to articles for specific communities such as clinical data (MTBLS2130, https://www.ebi.ac.uk/metabolights/MTBLS2130) (30) and plant data (MTBLS2876, https://www.ebi.ac.uk/metabolights/MTBLS2876) (14). This diverse pattern of data reuse highlights the value of public and FAIR data and MetaboLights welcomes further interactions with the community to continue such beneficial efforts.

## Technical development

The MetaboLights study repository has seen unprecedented growth in both the number of studies and the average size of studies in recent years. This presents significant technological challenges and compels MetaboLights to modernize current applications in an ongoing fashion as well as to develop new ones as requirements evolve.

Owing to EMBL-EBI’s data management and security policy, we have had to re-architect the process through which submitters upload data to MetaboLights. It is now a two-stage process, where data uploaded via FTP or Aspera must then be synchronized to the study directory. Simultaneously, in an effort to improve the user experience, metadata files and data files are now stored on separate volumes. This enhances updating and loading times of metadata files and facilitates easy organization of study files into interpretable categories. We have also recently implemented a limit (of two) on the number of studies a submitter can have in the ‘submitted’ state so to avoid spurious study creation and focus curation help.

Another major development on MetaboLights is the separation of application deployments to improve performance requirements. MetaboLights applications were maintained as a bundle. After an architectural change effort, the MetaboLights web page and MetaboLights Online Editor are maintained and deployed independently to support scalability and increase availability. This has also enabled MetaboLights to migrate current applications to microservice-based architecture and develop applications to query the contents of all public metadata at once. It will be a major focus to ensure that the full depth of metadata stored can be queried easily and combination queries facilitated more effectively across studies moving forward.

## MetaboLights Labs

**Figure 1.**
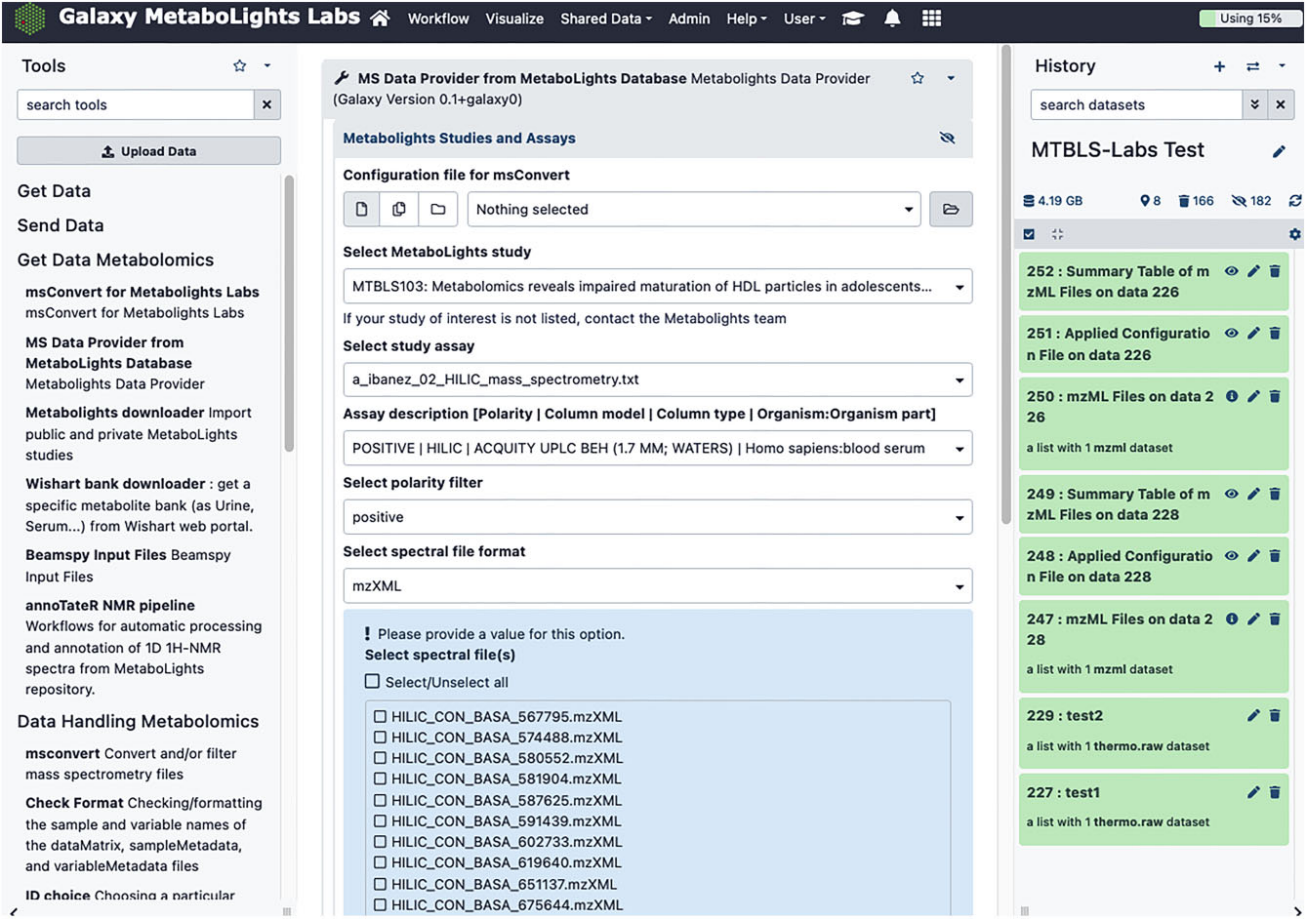
MetaboLights Labs website (https://metabolights-labs.org).

A complementary goal to providing the MetaboLights repository is to enable it to become a knowledgebase for the community. One aspect of this is the development of MetaboLights Labs (https://metabolights-labs.org), which is an open source and open access Galaxy Project (https://galaxyproject.org) instance (Figure 1). The aims are (i) to facilitate MetaboLights data reuse with high-quality analysis tools, (ii) to allow users to analyse their own data and (iii) to collaborate with researchers to contribute community tools and workflows.

MetaboLights Labs is the principal platform through which we expose bespoke computational workflows for the annotation of metabolites in both LC–MS and NMR datasets. These workflows are part of a general effort funded by the UKRI (UK Research and Innovation) to introduce repository-scale standardized workflows into the metabolomics analysis ecosystem.

### LC–MS tools and workflows

*MDP* (MetaboLights Data Provider) is a Galaxy Project tool wrapper on MetaboLights Labs to use MetaboLights LC–MS files in workflows. MetaboLights supports the most common open source derived and raw file formats (https://www.ebi.ac.uk/metabolights/editor/guides/Files/Raw_data). In the MDP tool, LC–MS studies, assays and files have been indexed so that users can filter data by study, assay, polarity and file type. Once users select MetaboLights LC–MS files, the MDP tool downloads, applies filters and converts the selected files to mzML format using msConvert (ProteoWizard) (31) with a predefined configuration file. It is also possible for users to define their custom msConvert configuration files and apply to MetaboLights LC–MS files. Finally, msaccess is executed to extract and summarize files/content.

*XCMS* is a framework that allows users to process chromatographic MS data (32,33). It contains different functions such as feature detection, alignment and correspondence to convert data files to data tables. The XCMS Galaxy Project tool wrappers (https://github.com/workflow4metabolomics/tools-metabolomics) (‘xcms_xcmsset’, ‘xcms_fillpeaks’, ‘xcms_retcor’ and ‘xcms_group’) have been deployed and standardized frameworks created to process MetaboLights LC–MS files.

*BEAMS* is a Python package that includes several automated and seamless computational modules that are applied to automatically annotate metabolites detected in untargeted LC–MS or DI–MS metabolomics assays. ‘BEAMSpy for Galaxy’ (https://github.com/computational-metabolomics/beamspy-galaxy) is a Galaxy Project tool wrapper of BEAMS and has been deployed on MetaboLights Labs to annotate MS1 data (features) from MetaboLights LC–MS files.

MetaboLights Labs provides optimized LC–MS workflows (Table 1) to use MetaboLights data or users’ own LC–MS data. Configuration parameters and files of these workflows have been optimized according to the requirements of current collaborators and we welcome feedback and further collaborators for future development.

**Table 1.** Optimized LC–MS workflows on MetaboLights Labs

#	Workflow name	Inputs	Workflow tools			
			MDP	msConvert	XCMS	BEAMSpy
1	XCMS Workflow for mzML Dataset	mzML dataset			X	
2	XCMS Workflow for mzML Dataset Collection	mzML dataset collection			X	
3	MetabolightsDB–XCMS Workflow	MetaboLights study	X	X	X	
4	MetabolightsDB–XCMS–BEAMSpy Workflow	MetaboLights study	X	X	X	X
5	myData–msConvert–XCMS–BEAMSpy	mzML dataset collection		X	X	X

Workflows have been applied to MTBLS2295 (https://www.ebi.ac.uk/metabolights/MTBLS2295), positive and negative, from MetaboLights as well as ST002571 from Metabolomics Workbench and MTBKS65 from MetaboBank with histories published (under ‘Shared Data’) on MetaboLights Labs (https://metabolights-labs.org/histories/list_published).

### NMR tools and workflows

*SAFER-NMR* is an R package that exposes for use a novel approach to annotation in 1D ^1^H NMR datasets (M.T. Judge *et al.*, unpublished data, 2023). It eschews traditional peaklists instead relying on extracting shapes and factoring in chemical shift, and statistically inferring relationships in the data. This means that annotation is not driven by expert knowledge, but by the tool itself, corroborated by evidence it generates to justify candidate annotations.

Publicly available tools for analysis of NMR datasets are few and far between, owing in part to the lack of easily accessible and reusable data. Approximately 97% of raw and derived spectral NMR data files in public studies are compressed, necessitating a download to work out interior structure and viability for reuse. To that end, we have developed a series of tools that allow for the indexing of spectral NMR data files, and the conversion of spectral data files into spectral data matrices. We have also developed the capability to compile reference files from data available on GISSMO (https://gissmo.bmrb.io).

While version 2 of the SAFER-NMR tool is still ongoing in its development, it is publicly accessible as an open source R package (https://doi.org/10.5281/zenodo.10022483), a docker image (https://hub.docker.com/r/mtbls/safer) and on MetaboLights Labs. The outputs of these tools for MTBLS1 (https://www.ebi.ac.uk/metabolights/MTBLS1), MTBLS395 (https://www.ebi.ac.uk/metabolights/MTBLS395), MTBLS424 (https://www.ebi.ac.uk/metabolights/MTBLS424) and MTBLS430 (https://www.ebi.ac.uk/metabolights/MTBLS430) are already available on MetaboLights’ public FTP server (http://ftp.ebi.ac.uk/pub/databases/metabolights/studies/mariana).

Also included in the package is a results viewer that allows for the user to interrogate the tool outputs in a granular fashion, down to single pieces of evidence for annotation. This represents a huge part of the value of the overall pipeline—not just generating matches, but being able to offer them up for expert visual scrutiny with ease.

## Outreach and training

MetaboLights is committed to support users offering online guides (https://www.ebi.ac.uk/metabolights/editor/guides), and online tutorials and courses (https://www.ebi.ac.uk/training/online/topic/metabolomics) to train researchers in using the resource effectively, along with providing assistance through metabolights-help@ebi.ac.uk. We encourage submitters to review the material outlined and of course will be happy to assist further. We welcome feedback on these help resources and ideas for future guides.

### Training courses and online webinars

As active members of the community, the MetaboLights team is also involved in initiatives that promote public awareness and community engagement, from delivering courses and webinars that introduce metabolomics to the general public and researchers of other disciplines, to leading workshops at international conferences. As an example of such activities, ‘Introduction to metabolomics analysis’ is a popular and oversubscribed annual training course. It provides an introduction to publicly available data, community standards, software and tools used for metabolomics data analysis and sharing, with a particular emphasis on the EMBL-EBI’s MetaboLights repository and Galaxy Project infrastructure. It is delivered through a mixture of presentations and practical hands-on use cases, to help attendants advance their skills in the analysis of metabolomics data. In 2023, this training was delivered in person at EMBL-EBI, in Hinxton, UK. From the 26 applicants selected, 77% were from a European country and 23% from other world countries (USA, India, Australia, Mexico and Singapore). Different career stages were represented among the attendants, who belonged to the academic and research and industry sectors. At the end of the training, 87% of participants provided an overall rating of ‘very good/excellent’, with a 100% responding positively when asked whether they intended to use the tools/resources covered in future work.

### Community feedback

**Figure 2.**
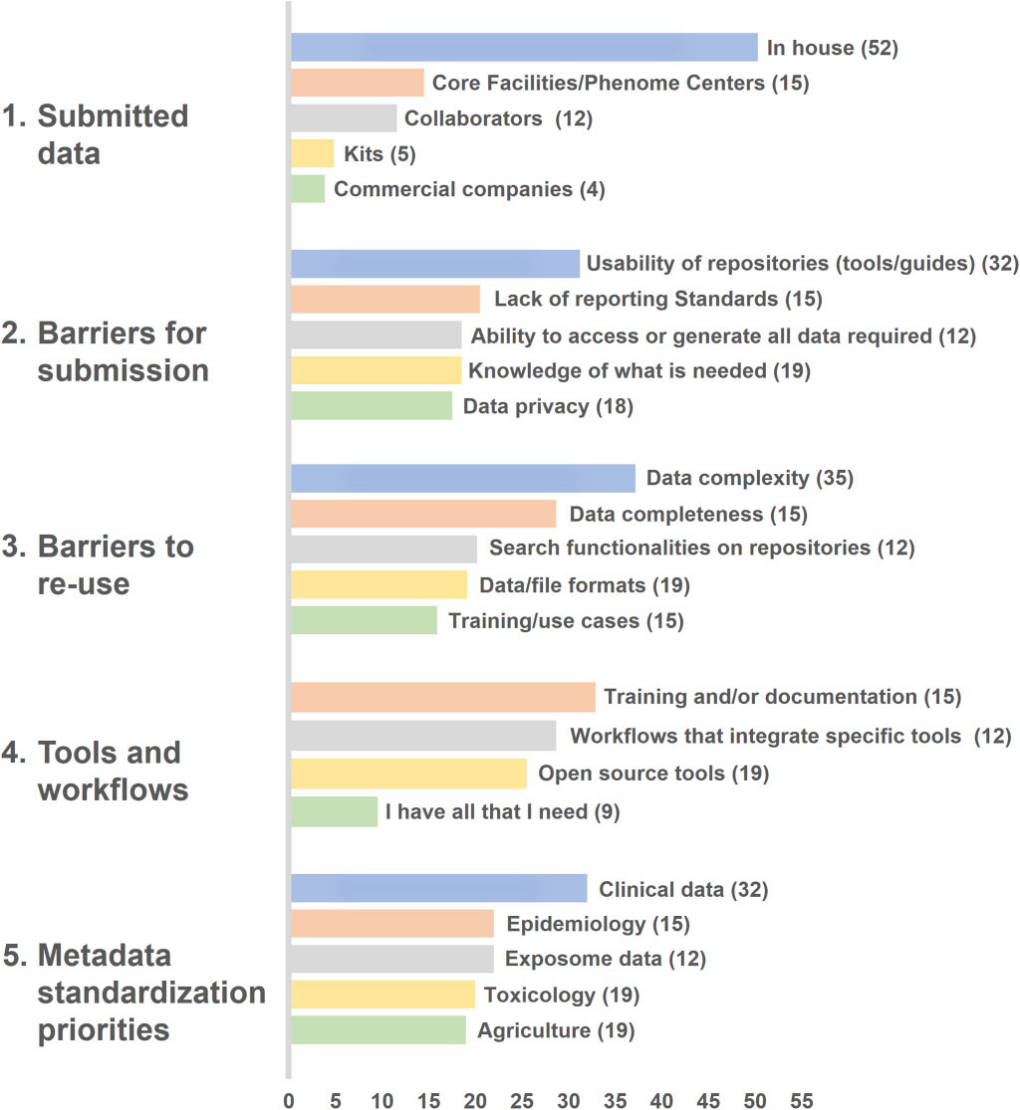
Results of the multiple-choice live survey conducted at Metabolomics 2023 (Niagara Falls, Canada). Values in parentheses represent the number of participants who selected that option.

In addition to face-to-face and online training, and in an effort to address the need for repositories and the metabolomics community to reinvigorate standards and collaborations, the MetaboLights team has organized outreach events at conferences such as the American Society for Mass Spectrometry (ASMS) 2023 Annual Conference in Houston, TX, USA, and Metabolomics 2023 in Niagara Falls, Canada. The ASMS 2023 workshop ‘From data to biology: using ’omics datasets to generate an unbiased hypothesis’ was focused on understanding the quality of MS data output and reducing the complexity of biological data extracted, with some of the most used and accepted workflows for interpreting an ’omics dataset and how best to approach data representation being presented. The most commonly used and freely available software to interpret proteomic data and display data graphically were also discussed, along with the repositories used to match newly generated data with pre-existing knowledge on gene/protein/post-translational modification data and how to cross-validate novel findings with what is reported in the literature. A final roundtable discussion of the most common issues and bottlenecks in data interpretation was held, with the overall goal of encouraging non-experts in bioinformatics to explore user-friendly resources for MS data analysis, at Metabolomics 2023, the workshop ‘Data Standardization and Reuse through Public Repositories’, where the providers of the major public metabolomics repositories (Metabolomics Workbench, MetaboBank and MetaboLights) presented the current state-of-the-art guidelines for experimental design, including protocols, metadata and results. The discussion was directed towards ways to create synergy among researchers and other resources through data reuse and standardization. In order to enable the repositories to gather feedback and understand the requirements of the community, a live survey (Figure 2) was used as part of the workshop. The survey will be revised and disseminated online to encourage greater granularity and better drive MetaboLights forward.

## Conclusion

It is an exciting time in the metabolomics field and MetaboLights is committed to ensuring that the metabolomics data being generated globally are captured in the most FAIR manner in collaboration with our repository colleagues and the wider scientific community. This article has highlighted the ongoing work on data standards, interaction with experts and the provision of easy-to-use data processing and data submission tools that we hope will facilitate the metabolomics community in evolving further. While we will continue to deliver in these areas as guided by workshop feedback, we also intend to develop further collaborations to enhance the reusability of data stored and to work more closely with other resources to ensure a comprehensive and complementary ecosystem for metabolomics. This will involve the development of more ontologies, integration of data and a redevelopment of the MetaboLights interface to ensure comprehensive and intelligent querying across different data types and studies. Simultaneously, we will continue to focus on efforts such as HoloFood, where interoperability across multi-omics resources enables a diverse group of communities to work together for mutual benefit and scientific progress.

We greatly value feedback from our user community. Please send your feedback and suggestions to metabolights-help@ebi.ac.uk.

## Data Availability

MetaboLights resources and data are available under the EMBL-EBI’s Terms of Use at https://www.ebi.ac.uk/metabolights and under Apache 2.0 at https://github.com/EBI-Metabolights and https://doi.org/10.5281/zenodo.10022483.
